# A promising novel material-light-cured wound closure adhesives promote wound healing after pilonidal sinus open surgery: a Case Report

**DOI:** 10.3389/fbioe.2025.1635598

**Published:** 2025-09-23

**Authors:** Jiachun Ni, Yiheng Yang, Qinyuan Liu, Yu Deng, Renjie Zhou, Linyong Zhu, Ying Li, Zhenyi Wang, Changpeng Han

**Affiliations:** ^1^ Department of Coloproctology, Yueyang Hospital of Integrated Traditional Chinese and Western Medicine, Shanghai University of Traditional Chinese Medicine, Shanghai, China; ^2^ School of Biomedical Engineering, Shanghai Jiao Tong University, Shanghai, China

**Keywords:** pilonidal sinus, surgery, wound healing, adhesives, case report

## Abstract

**Background/objectives:**

Pilonidal sinus disease (PSD), a common sacrococcygeal infectious disease, is characterized by sacrococcygeal swelling, pain, sacrococcygeal midline pits, sinus containing hair. Surgery is the main treatment for PSD. Although closure surgery reduces wound healing time, it has a high recurrence rate. Open surgery is the most effective procedure for treating PSD with reduced recurrence rates, but at the cost of large wounds, prolonged healing times, cumbersome care and long-term inactivity. Therefore, how to balance postoperative recurrence with prolonged wound healing time is a critical issue for clinicians. Herein, we introduced a novel material called light-cured wound closure adhesives, which is characterized by strong tissue binding strength, high biocompatibility, prominent wound healing capability.

**Methods:**

We present the case of a 22-year-old male with PSD who underwent open surgery. The light-cured wound closure adhesives were first applied to postoperative wound. We observed wound healing status, wound Numeric Rating Scale (NRS) pain scores, adverse events including exudation, infection, allergy, meanwhile documented the use of analgesics during follow-up period.

**Results:**

The wound healed at postoperative day 37, much shorter than that reported in the previous literature. The NRS pain scores are low. Adverse events includes excessive exudation, pruritus, granulation hyperplasia.

**Conclusion:**

Light-cured wound closure adhesives showed excellent pro-healing capabilities and brilliantly solved the paradox between high recurrence rate and prolonged wound healing time of PSD.

## 1 Introduction

Pilonidal sinus disease (PSD), a common sacrococcygeal infectious disease, is characterized by sacrococcygeal swelling, pain, sacrococcygeal midline pits, sinus containing hair (Brown and Lund, 2019). PSD is more prevalent in young people, more in males than in females (Luedi et al., 2020; Luedi et al., 2021), with a rising incidence of 39.6/10^5^–56/10^5^ (Faurschou et al., 2024; Oetzmann von Sochaczewski and Godeke, 2021). The pathogenesis of PSD remains unknown and two primary hypotheses have been proposed. Karydakis posits that PSD occurs as a result of the synergistic interaction of three factors: hairs with barbs, external forces leading to hair penetration of the skin, and the fragility of the skin (e.g., softness, or scars) (Karydakis, 1992). Bascom believes that PSD is mainly caused by enlarged hair follicles forming folliculitis, while hair serves as a secondary factor, so the removal of hair follicles is the main purpose of the surgery (Bascom, 1980).

Currently, surgery is the main treatment for PSD, which is categorized into closure and open surgery. Although closure surgery reduces wound healing time, it has a high recurrence rate (Stauffer et al., 2018). Open surgery is the most effective procedure for treating PSD (Milone et al., 2021) with reduced recurrence rates (Stauffer et al., 2018), but at the cost of large wounds, prolonged healing times, cumbersome care and long-term inactivity (de Parades et al., 2013). Therefore, how to balance postoperative recurrence with prolonged wound healing time is a critical issue for clinicians to address.

Medical bio-adhesive refers to the glue with the function of biological tissue bonding, which as a new type of material, is characterized by ease of use and rapid operation, meanwhile serves multiple functions including hemostasis, closure, filling, and providing structural support. Currently, the clinically available bio-adhesive is mainly medical grade cyanoacrylate, which can be rapidly polymerized and cured into a film when encountering trace anionic substances or organic amines at room temperature, then bonded with the wound. However, it exhibits inherent defects of insufficient flexibility, susceptibility to fracture, and suboptimal biocompatibility, which restrict its broader clinical application (Li et al., 2017; Lang et al., 2014; Annabi et al., 2017).

Hydrogel is a material with high water content and similar physical properties to natural extracellular matrix, which has the most similar structural and mechanical properties of human soft tissues. It can provide a good self-healing environment, meanwhile form an effective physical barrier to quickly stop bleeding and close the wound, resulting in promoting wound repair and healing. Photosensitive hydrogel is a gel formed by polymerization and cross-linking of biomolecules (chitosan, hyaluronic acid, etc.) modified by photosensitive groups after light excitation. Photosensitive hydrogel is liquid before light excitation, and quickly cures into gel after photoexcitation, which can realize *in-situ* localization of irregular wounds with excellent controllability. However, the current photosensitive hydrogel predominantly relies on initiators to trigger free-radical polymerization via double bonds to form hydrogels. This technique, constrained by its free-radical initiation mechanism, exhibits several inherent limitations (Williams et al., 2005; Mironi-Harpaz et al., 2012): (1) Potential cytotoxicity of free radicals: Free radicals may induce DNA, RNA, protein, or amino acid cross-linking or oxidative damage, leading to cellular injury and reduced biocompatibility; (2) Insufficient adhesion: The free-radical cross-linking reaction is oxygen-sensitive, making it challenging to achieve complete curing when forming thin hydrogel layers on tissues. Consequently, these hydrogels exhibit weak tissue adhesion and are prone to detachment.

In view to the above technical barriers, we developed an innovative method for the preparation of hydrogels by “photoinduced coupling reactions” non-free radical photochemical cross-linking method (Yang et al., 2016; Bao et al., 2023). The “photoinduced coupling reactions” gel technique uses hyaluronic acid (HA), a natural component of the human body, as the biomacromolecule skeleton, and uses photosensitive small molecules to modify it to cause the release of active groups from photosensitive molecules when photoactivated, so as to quickly cross-link the hyaluronic acid macromolecules to form a gel. The released active groups are covalently bound to the amino group of the wound tissue, thus realizing the firm integration between the gel and the tissue, seamless bonding of various tissues and wounds.

Cell counting kit-8 assay found that our light-cured wound closure adhesives, so as to photosensitive hydrogels, exhibited no significant cytotoxicity (>90% cell viability) against L929 fibroblast cell line after 24 h of action at different concentrations (10, 100, and 1000 μg/mL) (Yang et al., 2016). Biomechanical experiments revealed that the hydrogel had a strong tissue binding strength (43 kPa), which is three times stronger than fiber glue (13 kPa) (Yang et al., 2016). Animal experiments showed that this photosensitive hydrogel could promote the healing of rat and rabbit skin model (Yang et al., 2016; Zhang et al., 2021). In rabbit and pig surgical tests, the hydrogel stopped high-pressure bleeding in cardiac incision and femoral artery incision within 30 s (Hong et al., 2019). Furthermore, the hydrogel could shorten the wound healing time of rat and pig oral mucosa repair models (Zhang et al., 2021b).

In this case, we introduced a patient with PSD undergoing open surgery. The light-cured wound closure adhesives had been applied to postoperative wound with favorable results. Our photosensitive hydrogel brilliantly solved the paradox between high recurrence rate and prolonged wound healing time of PSD. The detailed information was reported as follows.

## 2 Case report

### 2.1 Patient information

A 21-year-old man was admitted to the Department of Coloproctology, Yueyang Hospital of Integrated Traditional Chinese and Western Medicine, Shanghai University of Traditional Chinese Medicine (Shanghai, China) due to recurrent sacrococcygeal swelling for a year. The patient underwent open drainage in another hospital 1 year ago because of sacrococcygeal abscess. He denied any history of other diseases, infectious diseases, surgery, trauma, and allergies.

### 2.2 Physical examination

**FIGURE 1:**
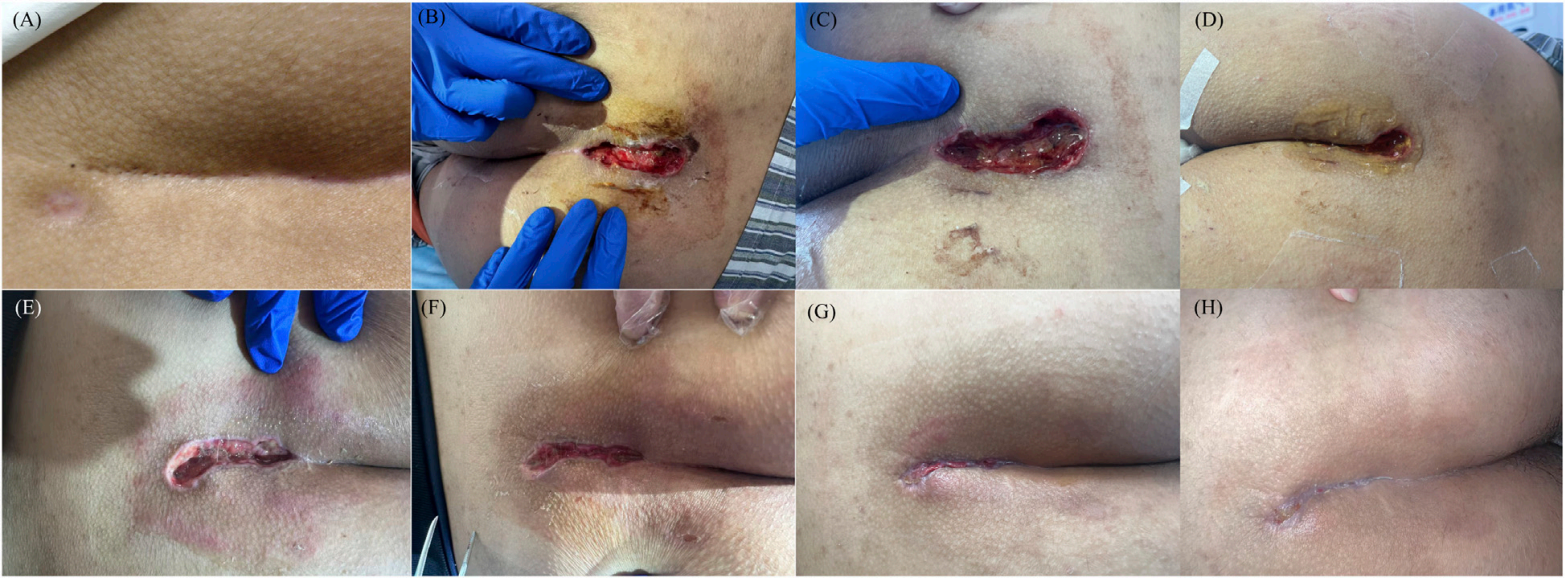
Wound status at each follow-up point. **(A)** Pre-operation; **(B)** Postoperative day1; **(C)** Postoperative day2; **(D)** Postoperative day3; **(E)** Postoperative day7; **(F)** Postoperative day14; **(G)** Postoperative day28; **(H)** Postoperative day37.

The sacrococcygeal region swelling and pain were positive. Eleven pathologic dimples were visible in the midline of the sacrococcygeal region (Figure 1A). Entering through the uppermost notch with a probe, a sinus tract about 5 cm long traveling toward the anus can be explored.

### 2.3 Imaging examinations

**FIGURE 2:**
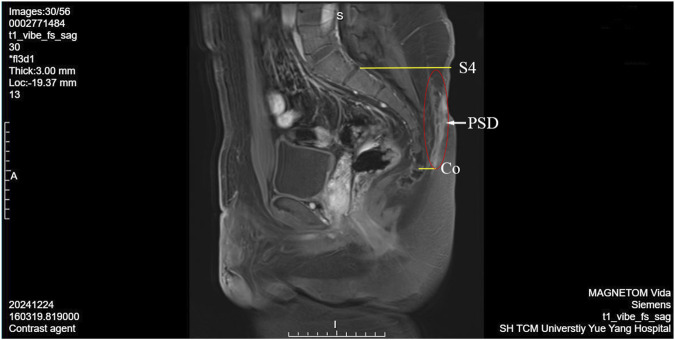
PSD enhanced sacrococcygeal MRI and hydrography image. S4, sacral vertebra 4; Co, coccygeal vertebra; PSD, pilonidal sinuses disease.

The patient underwent enhanced sacrococcygeal MRI and hydrography. Radiological manifestations: An irregular and abnormal lesion was found in the subcutaneous region of sacrococcygeal region (about the level of sacral vertebra 4 to coccygeal vertebra 1), while went from posterior upward slanting to anterior downward. The wall of the sinus tract showed low signal intensity on T1WI and slightly high signal intensity on T2WI. The fluid in the sinus tract showed low signal intensity on T1 and high signal intensity on T2. The sinus wall exhibited significant enhancement following the contrast-enhanced scan (Figure 2).

### 2.4 Laboratory examination

Preoperative blood routine, liver and kidney function and coagulation function showed no significant abnormalities. However, it was noteworthy that his progesterone and prolactin were high (0.33 ng/mL, 351 mL U/L respectively). Based on his symptoms and physical signs, he was diagnosed with pilonidal sinus.

### 2.5 Therapeutic intervention

#### 2.5.1 Surgery detail

An informed consent for surgery was signed by the patient preoperativly. PSD open surgery manipulation: After the administration of intravenous anesthesia, the sinus tract was initially explored using a probe and a curved vascular forcep. Subsequently, under the guidance of probe, the sinus tract was dissected layer by layer, and the purulent necrotic tissue was carefully debrided.

#### 2.5.2 Light-cured wound closure adhesive implementation

After the operation, the wounds were rinsed and sterilized with metronidazole solution and dried with sterile gauze. Then, the light-cured wound closure adhesive was evenly applied to the wounds, and the matching photosensitive hydrogel photocuring machine was used to irradiate the light-cured wound sealant for 30 s to achieve complete cure. At this time, the light-cured wound closure adhesive was a flat colloid, which was completely covered the surgical wound. Finally, the wound was covered with sterile gauze.

#### 2.5.3 Postoperative dressing change and follow-up

**FIGURE 3:**
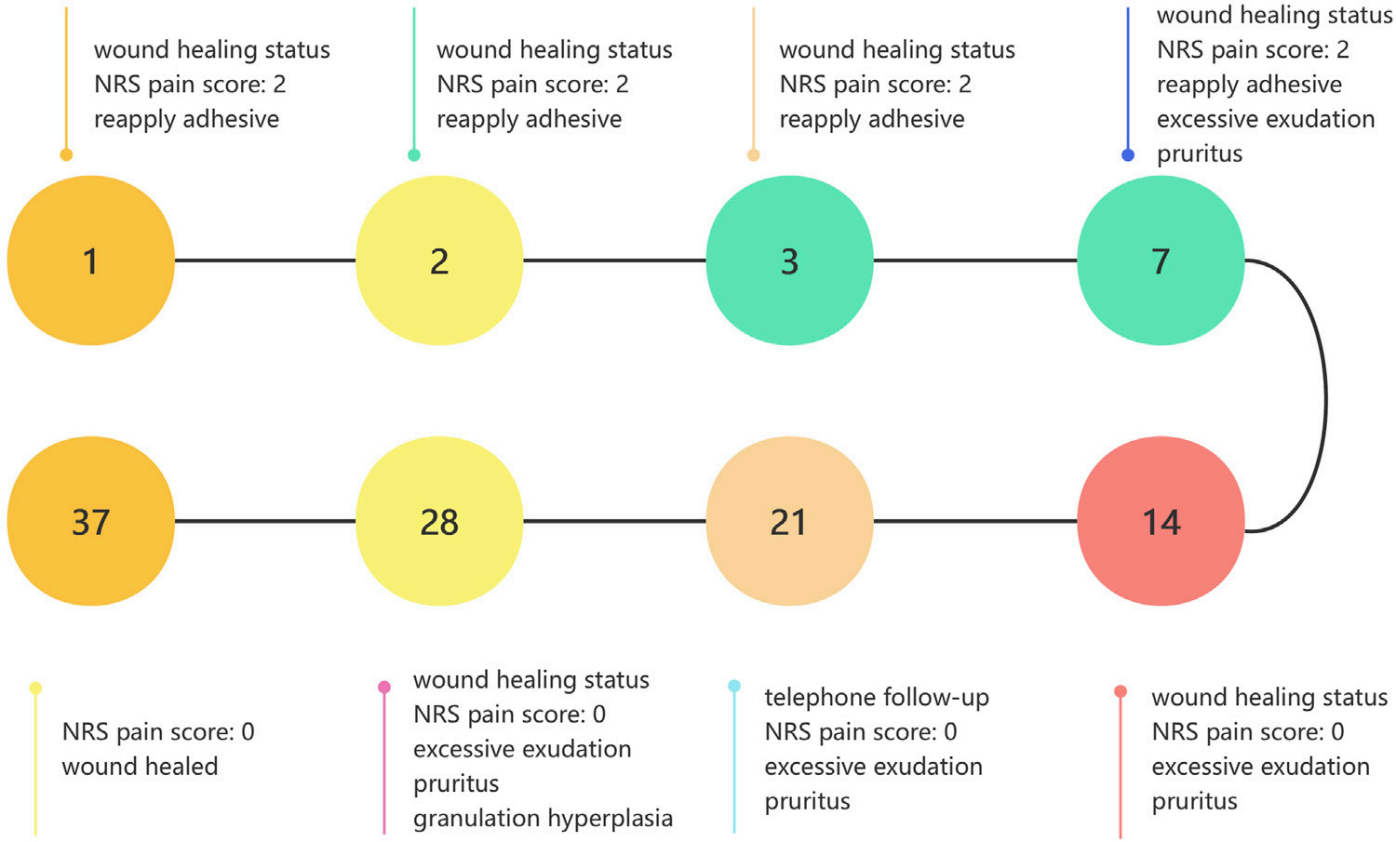
Brief timeline of wound healing. NRS, Numeric Rating Scale.

The brief timeline of wound healing was depicted in Figure 3. We assigned dedicated personnel to monitor the wound of patient at 9:00 a.m. from the first to the seventh postoperative day. In cases where the covering colloid detached, the wound was disinfected using hydrogen peroxide cotton balls, followed by reapplication of adhesive for curing. After the adhesive cured, two pieces of sterile gauze were placed over the wound and secured with tape. If the covering colloid remained intact, any stains could be gently removed by wiping around the wound with saline cotton balls. Seven days post-surgery, if the covering colloid detached, we wouldn’t reapply the adhesive. The wound would be disinfected with hydrogen peroxide cotton balls every morning and evening. The patient was instructed to abstain from using any medication or treatment that could potentially interfere with wound healing during the follow-up period.

We established eight follow-up points, viz. postoperative days 1, 2, 3, 7, 14, 21, 28, and 35. During the follow-up period, we observed wound healing status, wound Numeric Rating Scale (NRS) pain scores, adverse events including exudation, infection, allergy, meanwhile documented the use of analgesics. Wound healing was defined as 95% epithelialization.

## 3 Outcome

### 3.1 Wound healing

During the follow-up period, light-cured wound closure adhesive was well tolerated and patient exhibited a good adherence. Wound healing status at each follow-up time point was presented in Figures 1B–H. On postoperative day 21, the patient was unavailable for outpatient follow-up, so we conducted a telephone follow-up. On postoperative day 35, the patient was unable to attend the follow-up visit for personal reasons. Therefore, we assessed the wound healing status during the follow-up visit on postoperative day 37 as an alternative. Surprisedly, the wound had healed at postoperative day 37, which was significantly shorter than 54.64 ± 14.87 days and 62.20 ± 5.18 days recorded in previous literature (Song et al., 2022; Li et al., 2020).

### 3.2 NRS pain score and analgesic drugs usage

The NRS pain scores were 2 on postoperative days 1, 2, 3, and 7, then decreased to 0 in remaining follow-up period. From postoperative days 1 until wound healing the patient did not use analgesic drugs.

### 3.3 Adverse events

On postoperative days 7, 14, 21, and 28, the patient reported excessive exudation accompanied by pruritus. Therefore, we prescribed the patient mizolastine sustained-release tablets (Sanofi Winthrop Industrie, Gentilly, France) once a day, 10 mg orally each time, to relieve the symptom. On postoperative day 28, granulation tissue hyperplasia was observed, and the excess granulation tissue was carefully debride (Figure 1G).

## 4 Discussion

### 4.1 Possible causes of PSD and suggestion for prevention

In this case, the patient had undergone sacrococcygeal abscess open drainage prior to the onset of the disease. Some studies have shown that abnormal periwound hair proliferation may occur during the wound healing process, apotentially attributable to the increased levels of local epidermal growth factor (Vathananai and Lekhavat, 2022; Hyun et al., 2016). We hypothesize that the underlying causes of this condition are as follows: 1) Localized hair hypertrichosis, wherein the movement causes hair tips pierce into the skin following inflammatory responses; 2) During the healing process of the prior surgical wound, hair may have infiltrated the wound, leading to foreign body reactions and subsequent inflammation. Accordingly, we suggest that individuals with a history of PSD or hirsutism regularly get hair removal in the sacrococcygeal region.

### 4.2 Possible mechanism of light-cured wound closure adhesive

The primary ingredient in the Light-cured wound closure adhesive is HA. HA serves as a crucial component of the extracellular matrix and plays an essential role in the process of wound healing (Kim et al., 2019). During hemostasis phase, high molecular weight HA binds to the CD44 receptor on the platelet surface, thereby promoting platelet activation, aggregation, and thrombosis, thus achieving hemostasis effect (Frost and Weigel, 1990). During the inflammatory phase, high concentrations of HA activate tumour necrosis factor-alpha, interleukin-1 beta and interleukin-8, and thereby recruiting inflammatory cells to wound (Chen and Abatangelo, 1999; Hart, 2002). On the other hand, tumor necrosis factor-stimulated gene-6 expressed in fibroblasts stimulated by tumour necrosis factor-alpha and interleukin-1 binds to high molecular weight HA polymers can inhibit neutrophil migration and prevent inflammation by inhibiting plasmid via a negative feedback loop (Wisniewski and Vilcek, 1997; Fries and Kaczmarczyk, 2003). During the proliferative phase, high molecular weight HA can significantly upregulate interleukin-1 beta, interleukin-8 and vascular endothelial growth factor, as well as matrix metalloproteinase-9 and matrix metalloproteinase-13 in keratinocytes, meanwhile increase HaCaT cell proliferation and migration (Kawano et al., 2021).

### 4.3 Possible causes of adverse reactions

During the healing process, the patient complained about wound exudation and granulation proliferation. Wound exudate is a normal phenomenon in the process of wound healing. It occurs as a result of increased vascular permeability, induced by inflammatory mediators such as histamine and leukotrienes, leading to the exudation of liquid components from blood into the tissue space (World Union of Wound Healing Societies WUWHS Consensus Document, 2019). In the case of increased wound exudation, we believe it is related to the unique location of the wound. PSD is located in the sacrococcygeal region, the blood supply to this area is relatively poor, and it is susceptible to tension and lateral pressure when sitting (Harris and Holloway, 2012). Insufficient local perfusion is associated with increased exudation. Insufficient local perfusion can lead to hypoxia in the wound tissue, causing a large influx of neutrophils and macrophages, which release inflammatory factors and reactive oxygen species, resulting in a significant increase in capillary permeability and continuous exudation of plasma components, manifested as an increase in exudate volume (Trujillo et al., 2015; Guo and Dipietro, 2010). Otherwise, the wound is wrapped in clothing and dressing, creating a kind of localized confined space, which, together with mechanical friction on the wound from walking or sitting, may result in increased exudation. Pruritus is mostly caused by exudation. Therefore, we recommend that patients avoid prolonged sitting and walking, and wear breathable undergarments after surgery to facilitate recovery. For granulomatous proliferation, studies have shown that hydrogel promote the release of various growth factors, which stimulate the proliferation and differentiation of fibroblasts and promote granulation tissue formation. However, excessive release of growth factors may lead to excessive granulation tissue proliferation (Gu et al., 2023). Furthermore, the hypoxic environment created by hydrogel can stimulate neoangiogenesis, but excessive angiogenesis may trigger overgrowth of granulation tissue (Zhang et al., 2021c).

## 5 Conclusion and prospects

Through this case observation, we found that light-cured wound closure adhesive hold significant potential in facilitating wound healing after PSD open surgery. To further validate its clinical efficacy, we will conduct a randomized controlled trial. Meanwhile, we will deeply explore its healing-promoting mechanism. In conclusion, this case report presents a new material for the management of postoperative wounds following PSD open surgery and discusses the pathogenesis of PSD, the potential mechanism of action of hydrogel, and the causes of adverse reactions, thereby providing valuable insights for the work of medical hydrogel researchers and clinicians.

## Data Availability

The original contributions presented in the study are included in the article/Supplementary Material, further inquiries can be directed to the corresponding authors.
