# Supplementary material for: A promising novel material-light-cured wound closure adhesives promote wound healing after pilonidal sinus open surgery: a Case Report
Source: Front Bioeng Biotechnol. 2025 Sep 23;13:1635598. doi: 10.3389/fbioe.2025.1635598 (PMC12500601; doi:10.3389/fbioe.2025.1635598)
Supplement: Supplementary file 1 [file Supplementaryfile1.docx]

Supplementary Material

# Supplementary Figures and Tables


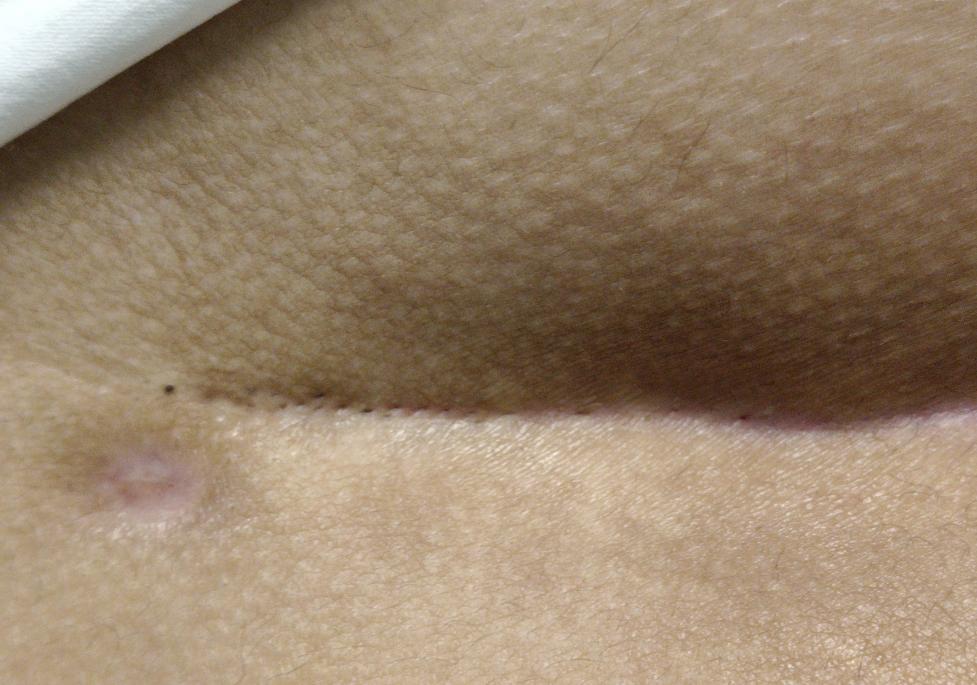


**Supplementary Figure 1.** Raw figure of Figure1A in manuscript.


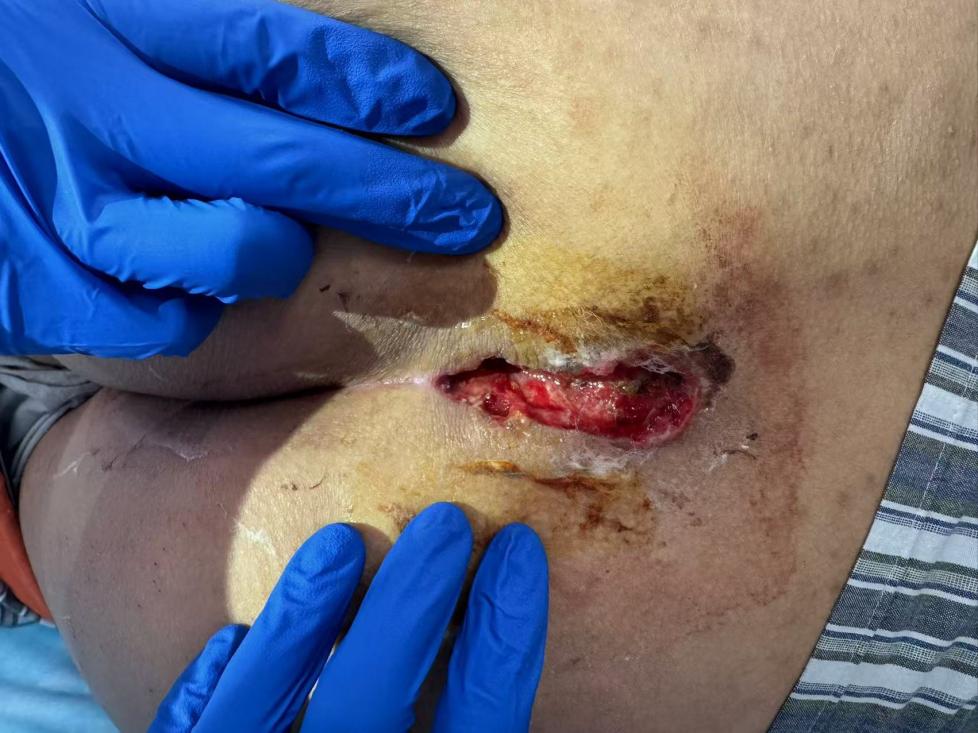


**Supplementary Figure 2.** Raw figure of Figure1B in manuscript.


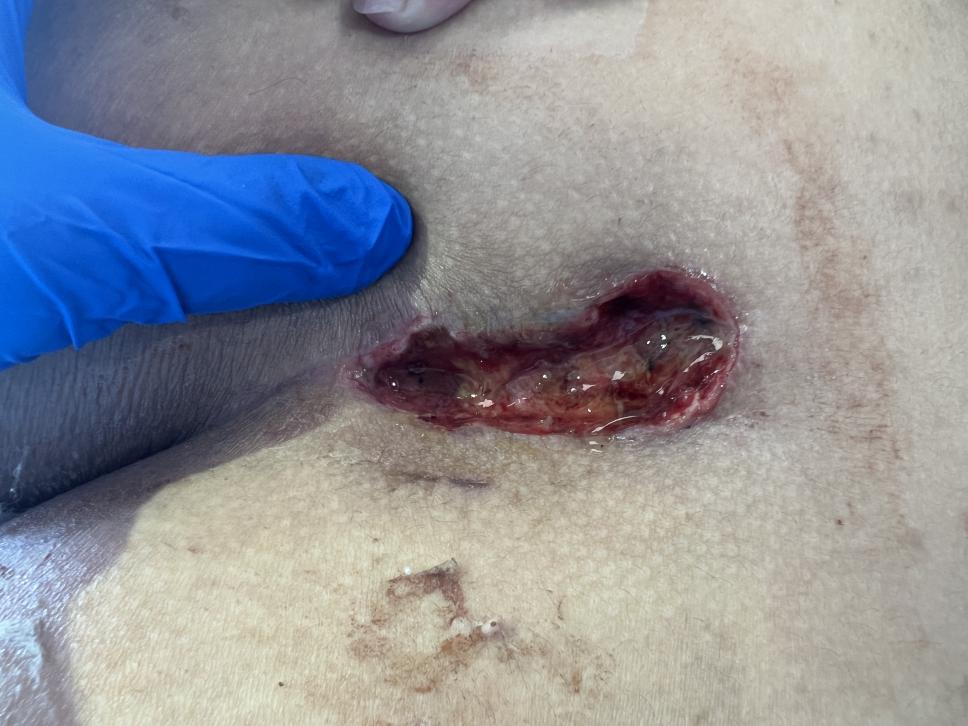


**Supplementary Figure 3.** Raw figure of Figure1C in manuscript.


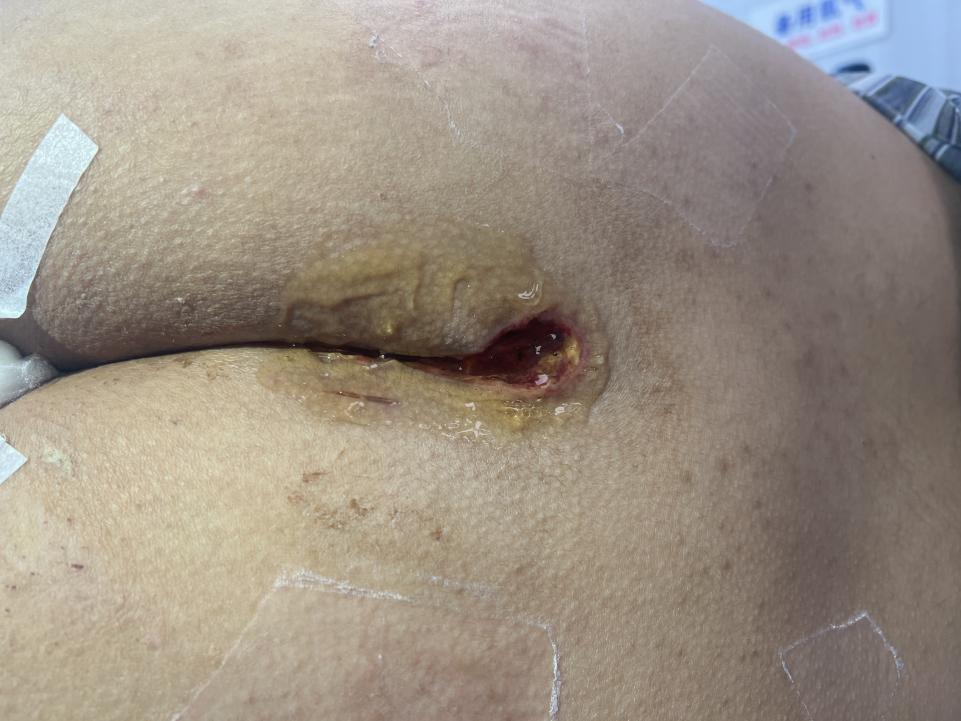


**Supplementary Figure 4.** Raw figure of Figure1D in manuscript.


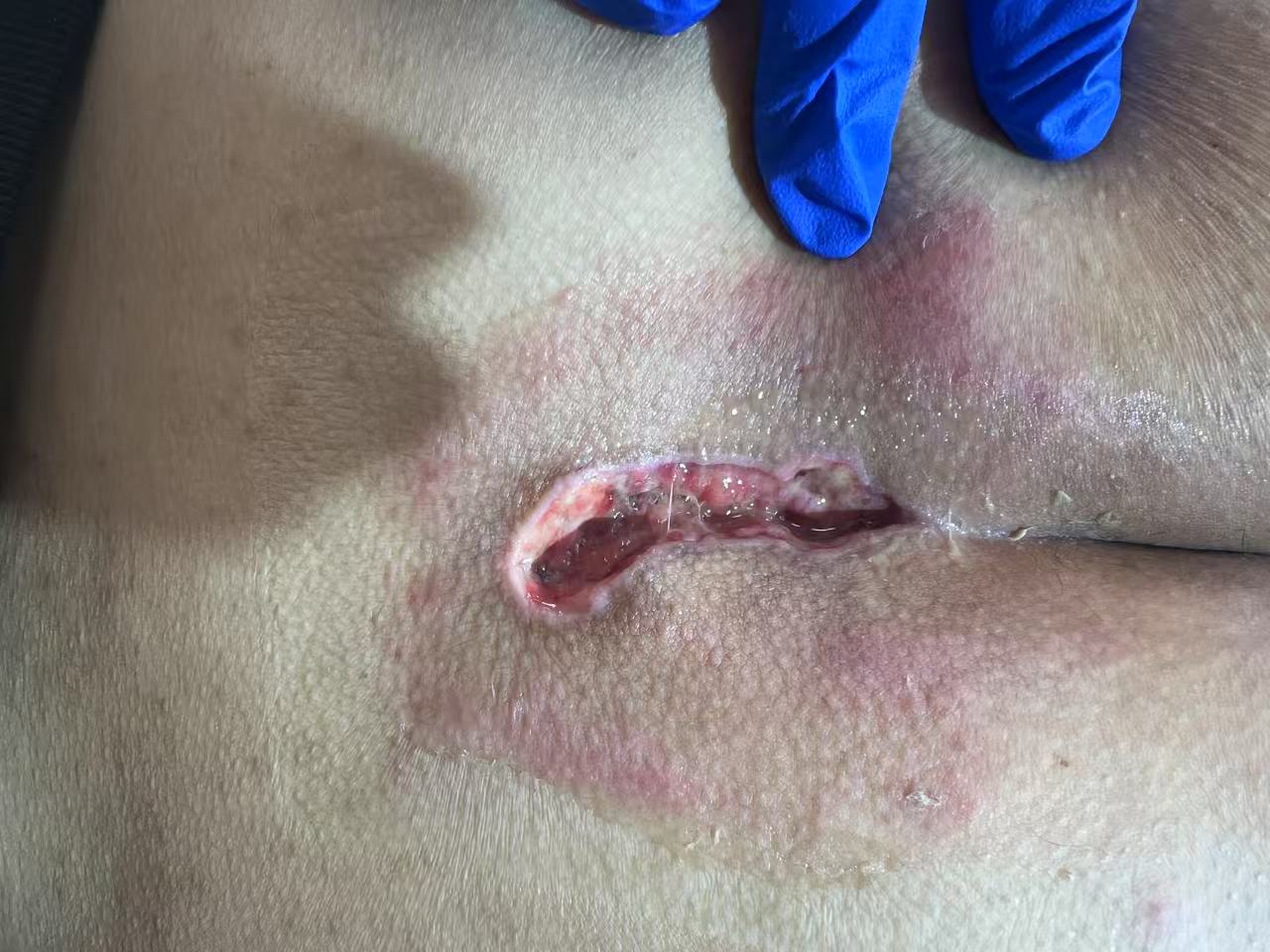


**Supplementary Figure 5.** Raw figure of Figure1E in manuscript.


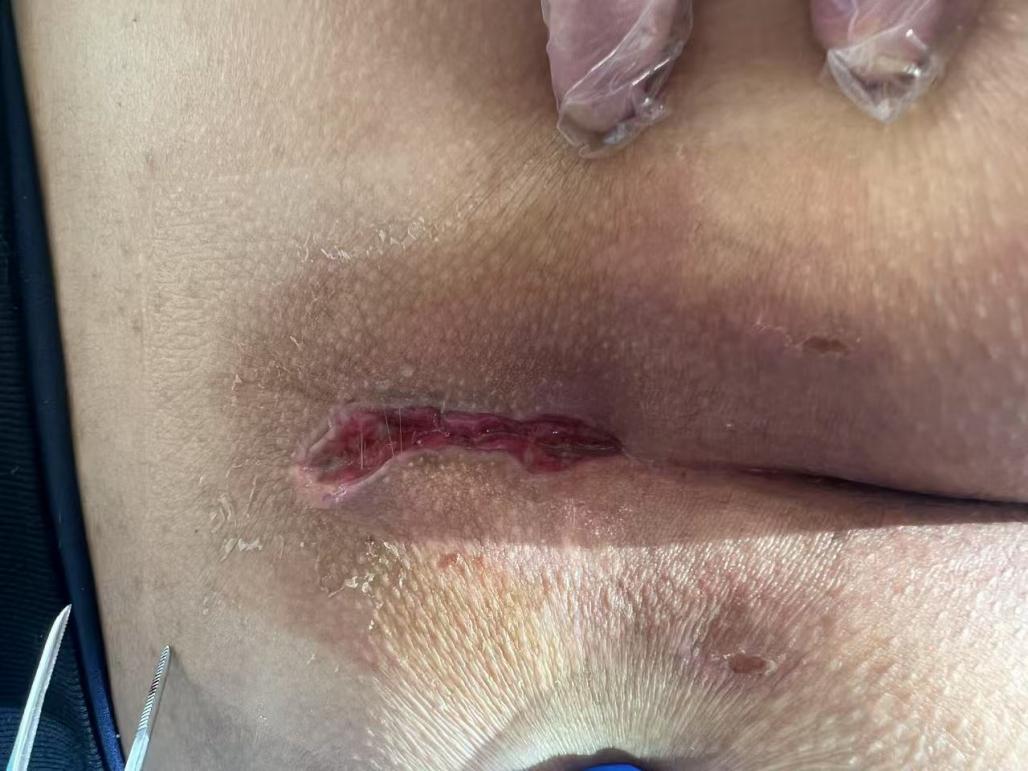


**Supplementary Figure 6.** Raw figure of Figure1F in manuscript.


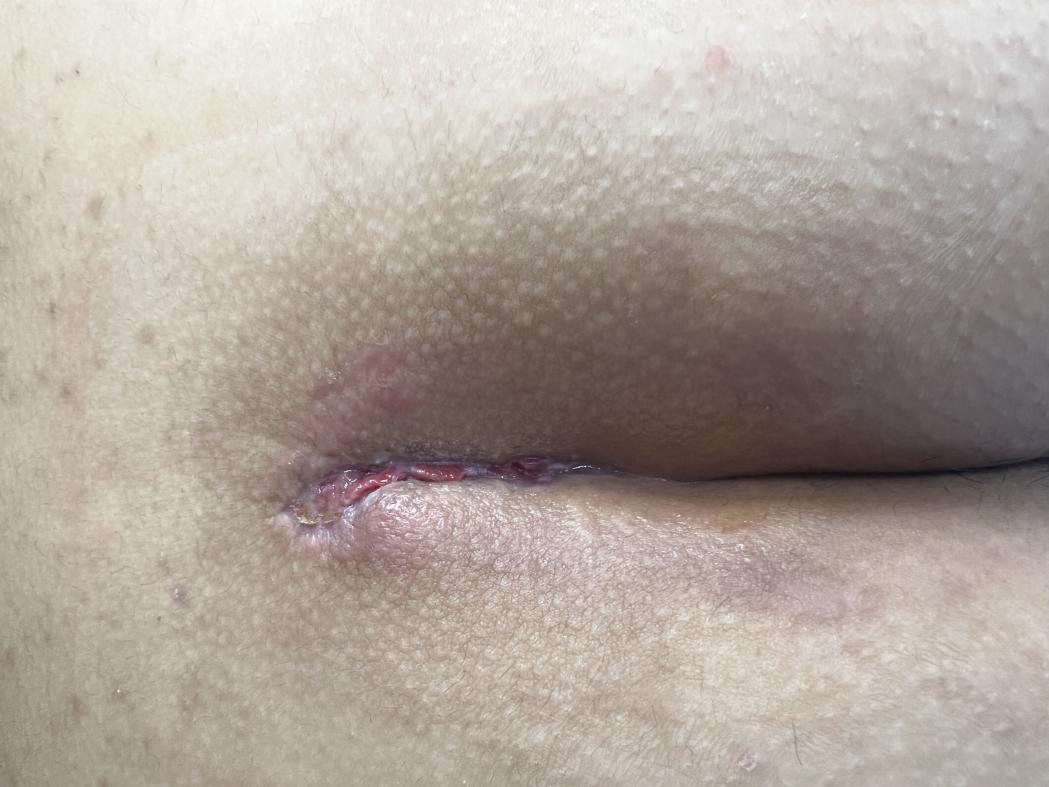


**Supplementary Figure 7.** Raw figure of Figure1G in manuscript.


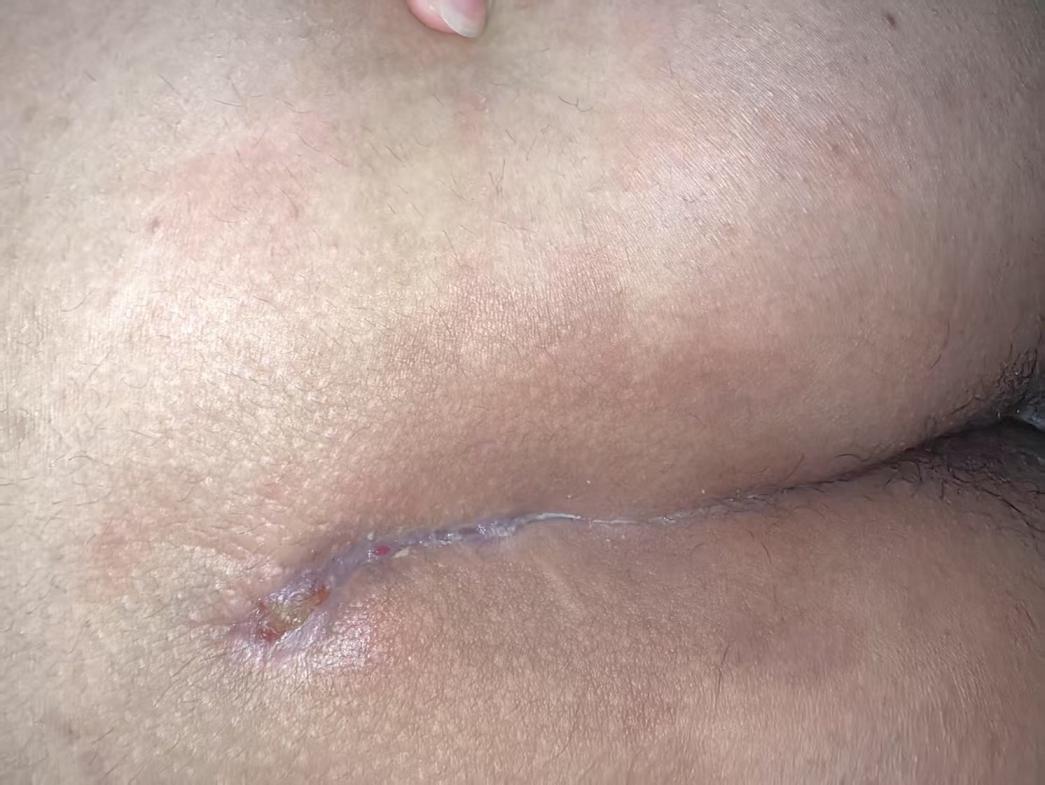


**Supplementary Figure 8.** Raw figure of Figure1H in manuscript.
